# Supplementary figures and images for: Cancer cell-derived interleukin-33 decoy receptor sST2 enhances orthotopic tumor growth in a murine pancreatic cancer model
Source: PLoS One. 2020 Apr 27;15(4):e0232230. doi: 10.1371/journal.pone.0232230 (PMC7185704; doi:10.1371/journal.pone.0232230)

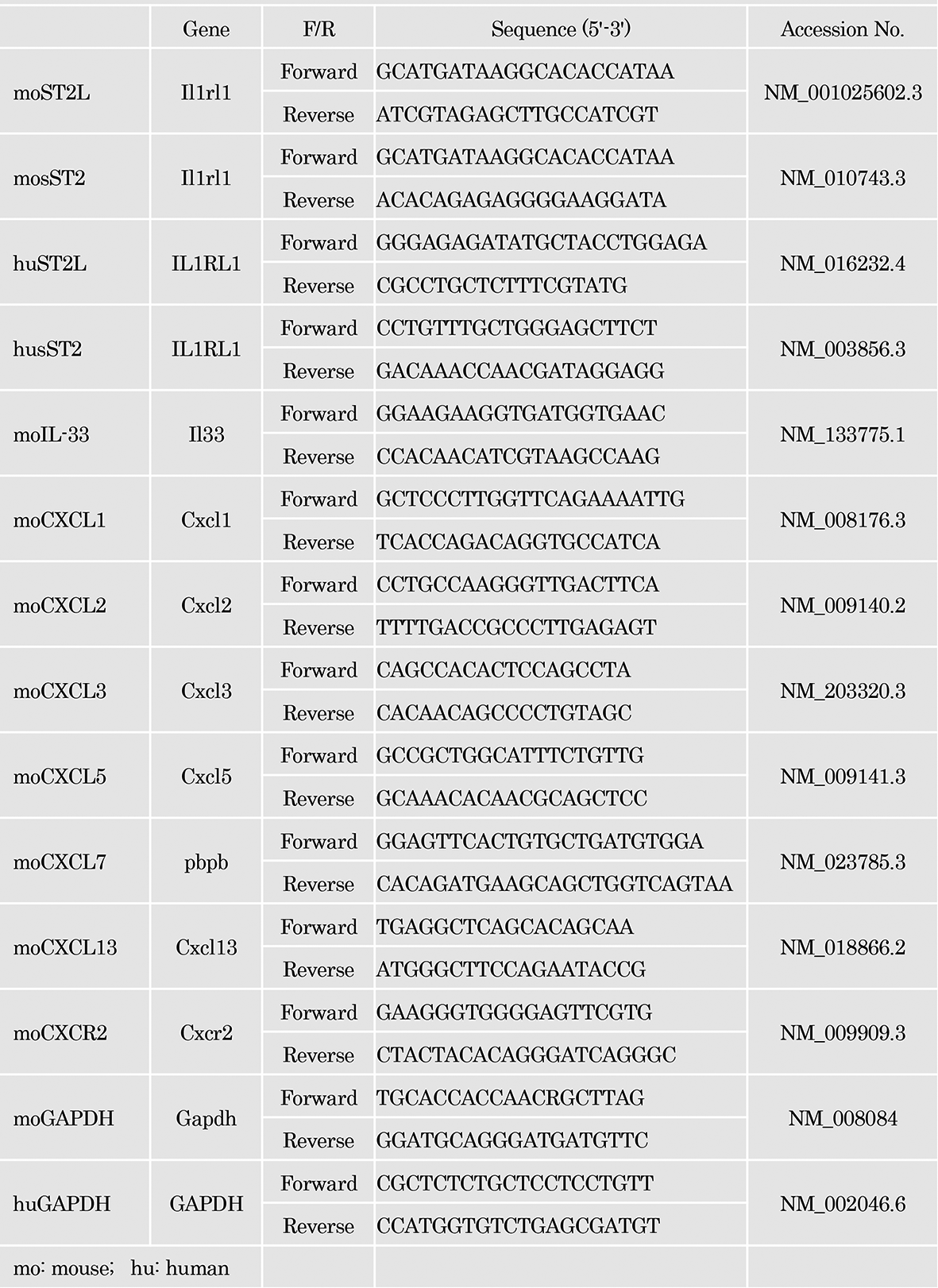

Supplement: S1 Table — (TIF) [file pone.0232230.s001.tif]

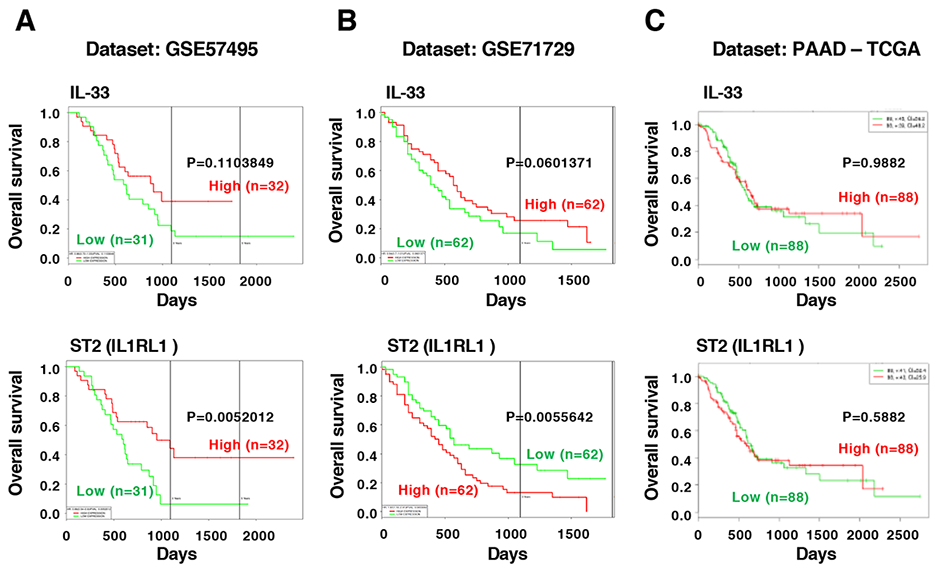

Supplement: S1 Fig — (TIF) [file pone.0232230.s002.tif]

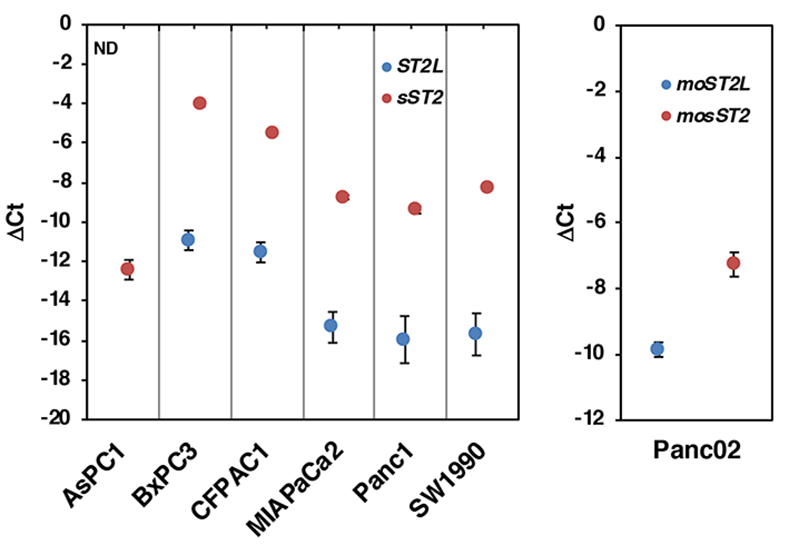

Supplement: S2 Fig — The expression level is expressed as ΔCt. (A) Human pancreatic cancer cell lines. (B) Mouse pancreatic cancer Panc02 cells. moST2L: mouse ST2L; mosST2: mouse sST2. ND: not detected. (TIF) [file pone.0232230.s003.tif]

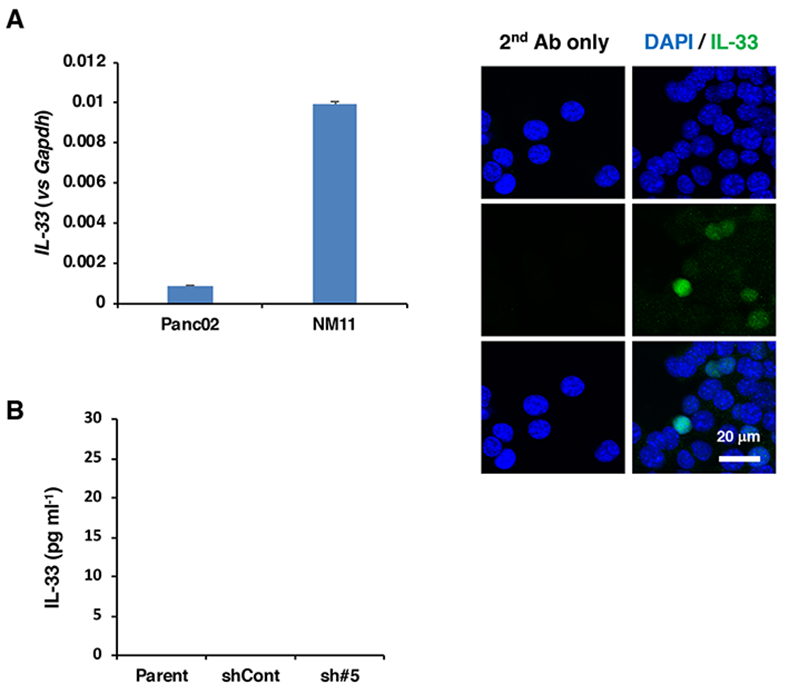

Supplement: S3 Fig — Expression and secretion of IL-33 in Panc02 parent and variant cells. (A) qRT-PCR analysis of the expression of IL-33 mRNA in Panc02 cells and mouse colon carcinoma NM11 cells (Nat Commun. 2016;7:13589) (left panel) in which IL-33 is localized in the nucleus (right panel). (B) Secretion of IL-33. Cells (4 × 10^4^ cells/100 μl) were cultured for 24 h, and the conditioned media were subjected to IL-33 ELISA analysis. The amount of IL-33 was below the detection limits. (TIF) [file pone.0232230.s004.tif]

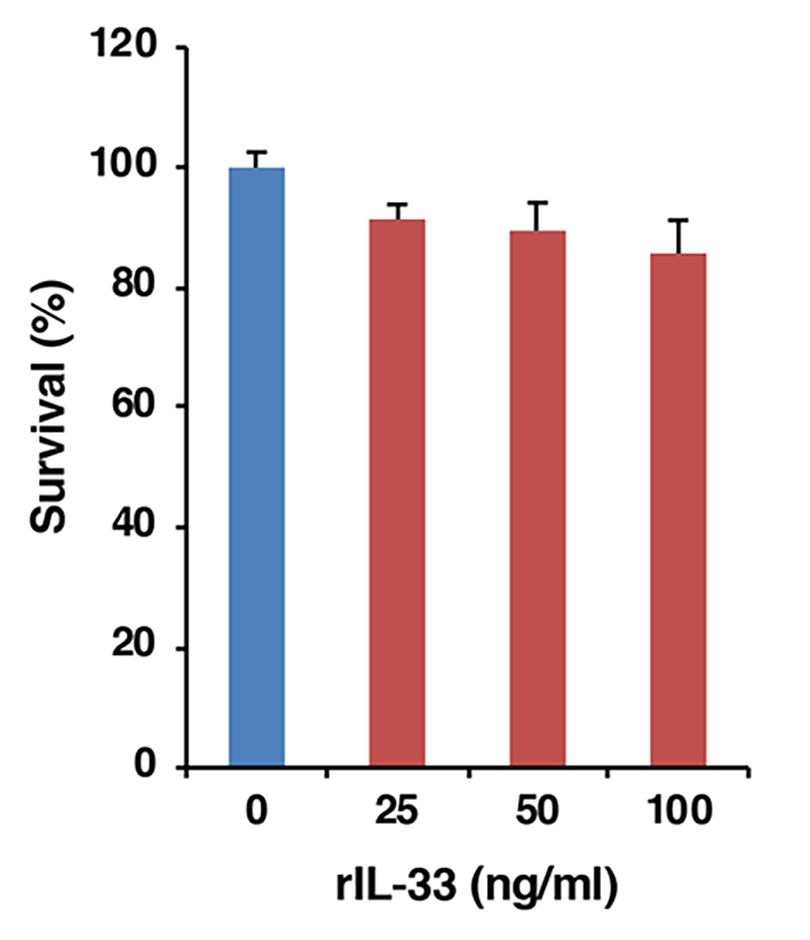

Supplement: S4 Fig — The cells were treated with the indicated concentrations of rIL-33 for 48 h. An MTT assay was used to measure cell survival. (TIF) [file pone.0232230.s005.tif]

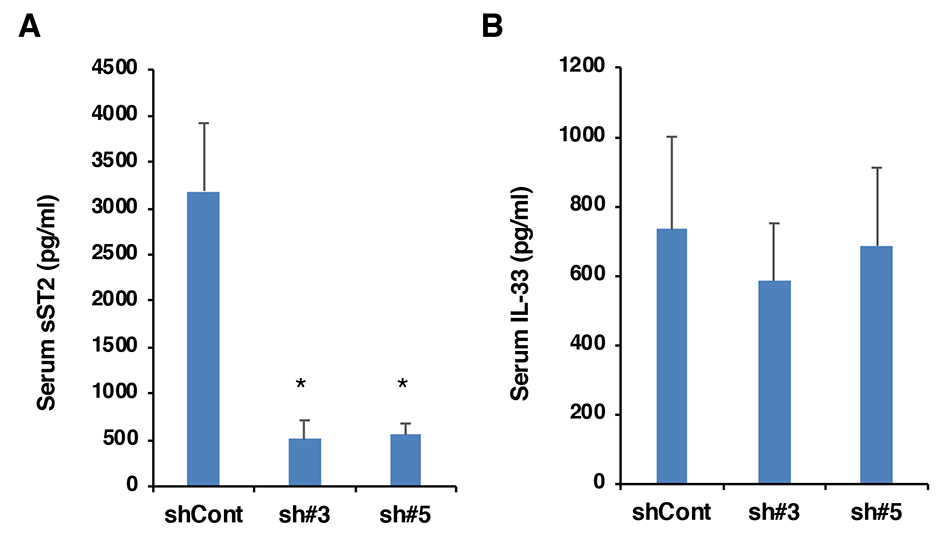

Supplement: S5 Fig — (A) sST2. (B) IL-33. Sera of the mice bearing shCont tumors (n = 5), sh#3 tumors (n = 4) and sh#5 tumors (n = 4) were used. *P<0.001 compared to shCont. (TIF) [file pone.0232230.s006.tif]

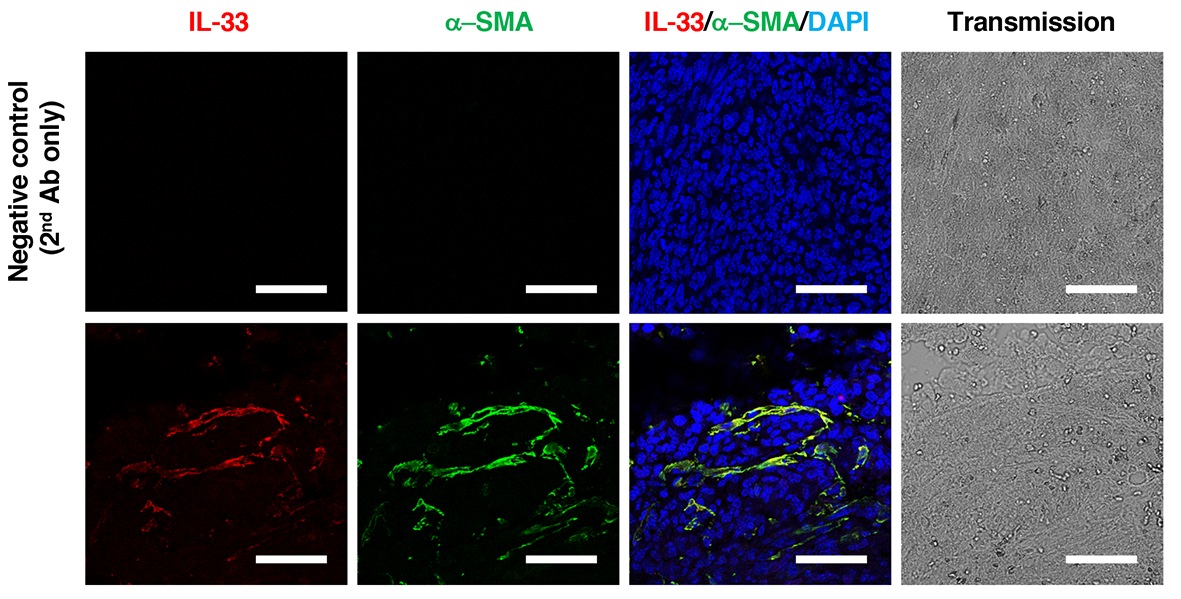

Supplement: S6 Fig — Sections of Panc02-shCont orthotopic tumors were immunostained for IL-33 and α-SMA. Bar: 50 μm. (TIF) [file pone.0232230.s007.tif]

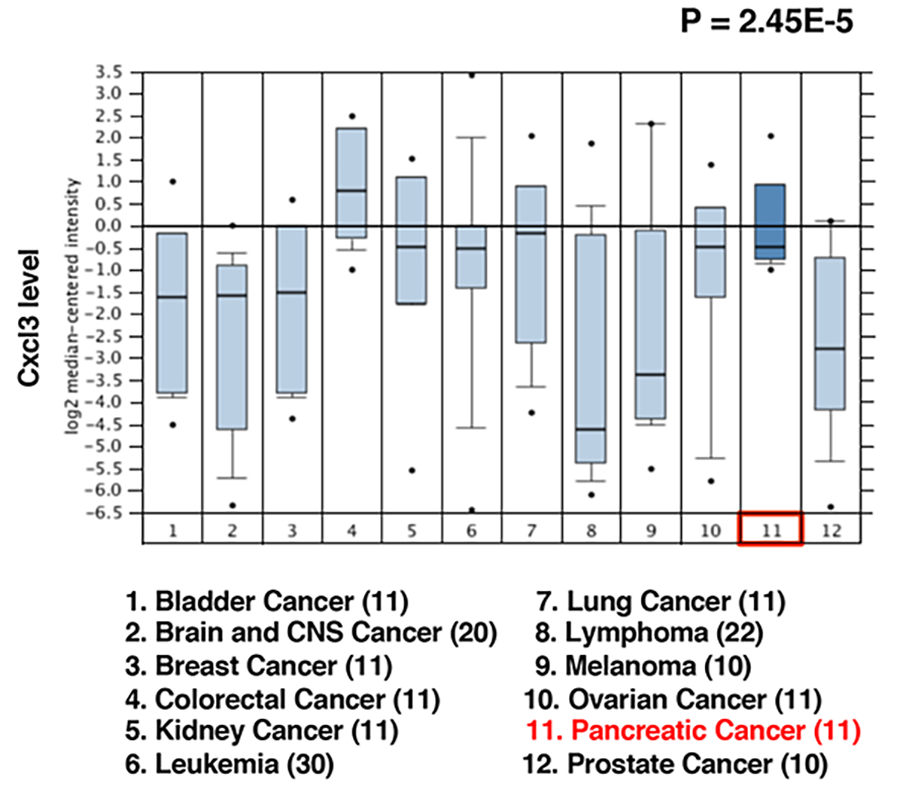

Supplement: S7 Fig — (TIF) [file pone.0232230.s008.tif]

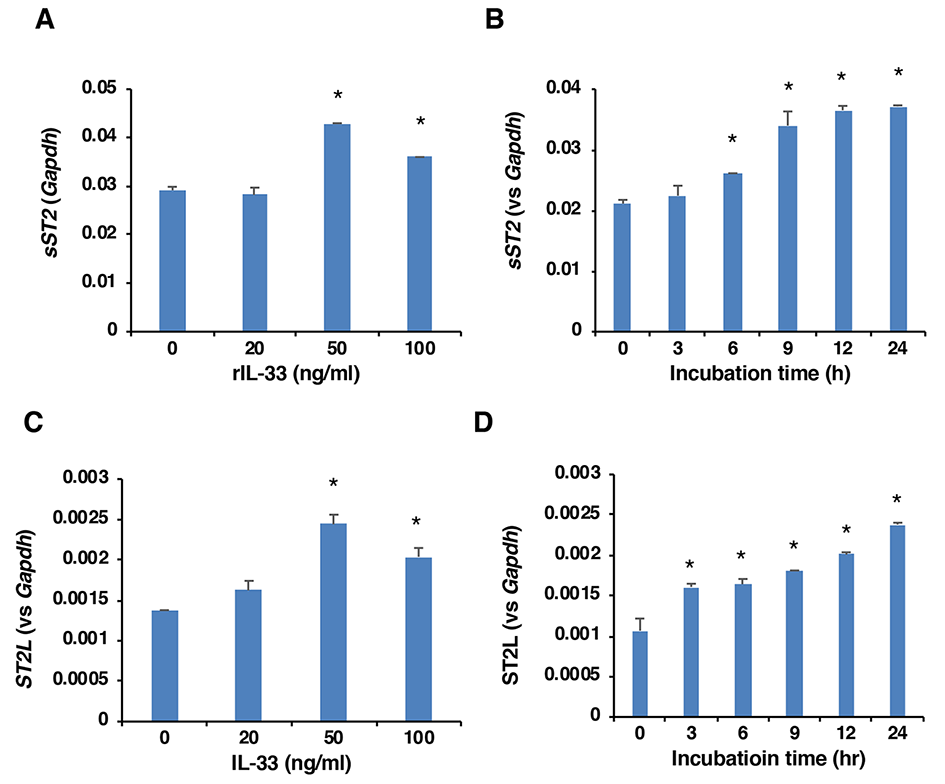

Supplement: S8 Fig — (A) The cells were treated with the indicated concentrations of rIL-33 for 6 h. (B) The cells were treated with 50 ng/ml rIL-33 for the indicated times. *P<0.05 compared to untreated cells. (TIF) [file pone.0232230.s009.tif]

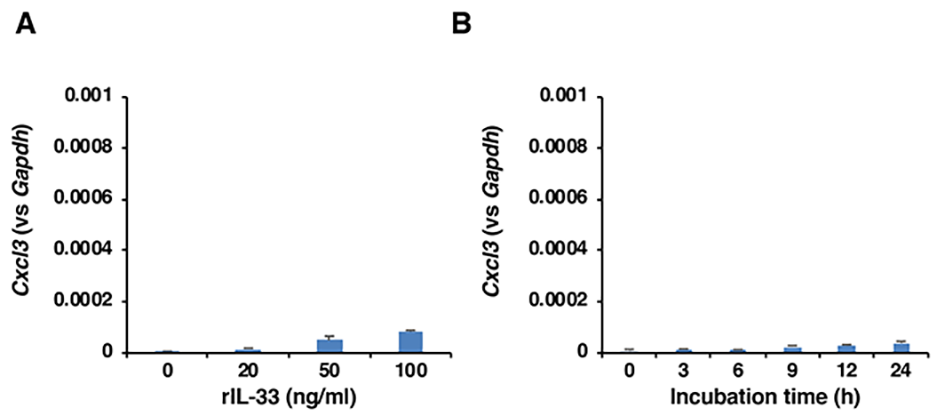

Supplement: S9 Fig — (A) The cells were treated with the indicated concentrations of rIL-33 for 6 h. (B) The cells were treated with 50 ng/ml rIL-33 for the indicated times. (TIF) [file pone.0232230.s010.tif]
