# Supplementary material for: Cancer cell-derived interleukin-33 decoy receptor sST2 enhances orthotopic tumor growth in a murine pancreatic cancer model
Source: PLoS One. 2020 Apr 27;15(4):e0232230. doi: 10.1371/journal.pone.0232230 (PMC7185704; doi:10.1371/journal.pone.0232230)
Supplement: Attachment — Submitted filename: plos one review.docx [file pone.0232230.s011.docx]

Here, Takenaga and colleagues conducted an *in vivo* study to explore the role of cancer cell derived IL-33 decoy receptor sST2 in pancreatic tumor growth. This is an interesting study as this one relatively new in pancreatic cancer and almost unexplored. The author precisely investigated the involvement of decoy receptor in tumor progression. However, in my opinion the manuscript has some shortcomings. Below I have provided numerous remarks. Please address the following concern.

Comments:

1. What is the source of IL-33 in TME. Is it pancreatic stellate cell or other cell types?

2. As a ligand, is IL-33 regulate the secretion of receptor sST2 from pancreatic cancer cell? If so, which control the secretion of receptor sST2 in global IL-33 KO mice. It is interesting to see that IL-33 signaling downregulate CXCL-3 expression, what could be the possible mechanism?

3. In figure3 panel E and F, is VC and sST2 are statistically significant?

4. It will be worthy, in future to perform some coculture experiment with PANC02 cell and CAF cell to better understand their cytokine/chemokine crosstalk in TME.
